# Predicting the return of spontaneous circulation after out-of-hospital cardiac arrest through blood gas analysis

**DOI:** 10.1186/2197-425X-3-S1-A206

**Published:** 2015-10-01

**Authors:** N Umei, I Shingo, Y Ujike, T Yumoto, A Ida, T Hirayama, N Shiba, K Tsukahara, Y Kinami, M Terado, H Yamanouchi, K Sato, T Ugawa

**Affiliations:** Department of Emergency Medicine and Critical Care, Okayama University Hospital, Okayama, Japan

## Intr

Several studies have investigated the factors associated with the return of spontaneous circulation (ROSC) in out-of-hospital cardiac arrest (OHCA) cases. The most analyzed factor is pre-hospital resuscitation, such as an early phone call for the ambulance, bystander cardiopulmonary resuscitation (CPR), presentation with ventricular fibrillation, and medication. The blood gas parameters have not yet been reported as a predictive factor for ROSC.

## Objectives

This study aimed to determine whether the blood pH, partial carbon dioxide pressure (PC02), bicarbonate (HC03^-^), hemoglobin, natrium, potassium, lactate, and glucose concentration on admission were able to predict ROSC in patients with OHCA.

## Methods

We performed a retrospective observational study of 186 OHCA patients who visited the emergency department of our hospital from January 2008 to December 2013. Bystander CPR status, initial cardiac rhythm, cause of arrest, the time from call receipt to hospital arrival, sex, age, and parameters of blood gas (pH, PC02,HC03^-^, hemoglobin, natrium, potassium, lactate, and glucose) were measured and compared between the ROSC-achieving patients (ROSC group) and those who did not achieve ROSC (non-ROSC group).

## Results

Among the OHCA patients, 67% did not achieve ROSC. The patients who did not achieve ROSC had a lower percentage of bystander CPR than those who achieved ROSC (13% vs. 31% p = 0.004). The PC02 and potassium levels on admission were significantly higher in the non-ROSC group (84 mmHg vs. 69 mmHg P = 0.03; 6.7 mmol/l vs. 5.2 mmol/l P < 0.01, respectively). The glucose concentrations were significantly lower in the non-ROSC group (149 mg/dl vs. 258 mg/dl p < 0.01). There were no statistically significant differences between both groups for other blood gas parameters. On multivariate analysis, bystander CPR as well as PC02, potassium, and glucose levels were independently associated with ROSC.

## Conclusions

A high blood PC02 level, a high potassium level, and low glucose level measured in OHCA patients on arrival at the hospital is predictive of a poor outcome for ROSC.
